# Glucocorticoid-induced leucine zipper (GILZ) is involved in glucocorticoid-induced and mineralocorticoid-induced leptin production by osteoarthritis synovial fibroblasts

**DOI:** 10.1186/s13075-016-1119-6

**Published:** 2016-10-04

**Authors:** Olivier Malaise, Biserka Relic, Edith Charlier, Mustapha Zeddou, Sophie Neuville, Céline Deroyer, Philippe Gillet, Edouard Louis, Michel G. Malaise, Dominique de Seny

**Affiliations:** 1 Laboratory of Rheumatology, Arthropôle, GIGA Research, University and CHU of Liège, Liège, Belgium; 2 Department of Orthopedic Surgery, CHU of Liège, Liège, Belgium; 3 Laboratory of Gastroenterology, GIGA Research, University and CHU of Liège, Liège, Belgium

**Keywords:** Leptin, GILZ, Osteoarthritis, Glucocorticoids, Mineralocorticoids, Synovial fibroblasts

## Abstract

**Background:**

Glucocorticoid-induced leucine zipper (GILZ) is a mediator of the anti-inflammatory activities of glucocorticoids. However, GILZ deletion does not impair the anti-inflammatory activities of exogenous glucocorticoids in mice arthritis models and GILZ could also mediate some glucocorticoid-related adverse events. Osteoarthritis (OA) is a metabolic disorder that is partly attributed to adipokines such as leptin, and we previously observed that glucocorticoids induced leptin secretion in OA synovial fibroblasts. The purpose of this study was to position GILZ in OA through its involvement in the anti-inflammatory activities of glucocorticoids and/or in the metabolic pathway of leptin induction. The influences of mineralocorticoids on GILZ and leptin expression were also investigated.

**Methods:**

Human synovial fibroblasts were isolated from OA patients during knee replacement surgery. Then, the cells were treated with a glucocorticoid (prednisolone), a mineralocorticoid (aldosterone), a glucocorticoid receptor (GR) antagonist (mifepristone), a selective glucocorticoid receptor agonist (Compound A), mineralocorticoid receptor (MR) antagonists (eplerenone and spironolactone), TNF-α or transforming growth factor (TGF)-β. Cells were transfected with shRNA lentiviruses for the silencing of GILZ and GR. The leptin, IL-6, IL-8 and matrix metalloproteinase (MMP)-1 levels were measured by ELISA. Leptin, the leptin receptor (Ob-R), GR and GILZ expression levels were analyzed by western blotting and/or RT-qPCR.

**Results:**

(1) The glucocorticoid prednisolone and the mineralocorticoid aldosterone induced GILZ expression dose-dependently in OA synovial fibroblasts, through GR but not MR. Similar effects on leptin and Ob-R were observed: leptin secretion and Ob-R expression were also induced by prednisolone and aldosterone through GR; (2) GILZ silencing experiments demonstrated that GILZ was involved in the glucocorticoid-induced and mineralocorticoid-induced leptin secretion and Ob-R expression in OA synovial fibroblasts; and (3) GILZ inhibition did not alter the production of pro-inflammatory cytokines by OA synovial fibroblast or the anti-inflammatory properties of glucocorticoids.

**Conclusions:**

The absence of GILZ prevents corticoid-induced leptin and Ob-R expression without affecting the anti-inflammatory properties of glucocorticoids in OA synovial fibroblasts. Mineralocorticoids also induce leptin and Ob-R expression through GILZ.

## Background

The glucocorticoid-induced leucine zipper (GILZ) protein is an intracellular protein that is induced by glucocorticoids. Recent studies have highlighted a major role of GILZ in the anti-inflammatory activities of glucocorticoids. GILZ inhibits the nuclear factor (NF)-kB pathway in human macrophage cells, epithelial respiratory cells and T lymphocytes [1–4]. GILZ also promotes T helper (Th)17 cell polarization toward a regulatory mesenchymal stem cell phenotype in arthritis [5], and it even displays additional functions in depression [6] and spermatogenesis [7]. Further, GILZ is expressed by synovial fibroblasts and is an endogenous anti-inflammatory mediator in rheumatoid arthritis [8]. Ngo et al*.* have reported the anti-inflammatory activity of exogenous GILZ in the treatment of collagen-induced arthritis [9]. However, GILZ depletion does not always impair the anti-inflammatory activities of exogenous glucocorticoids [9, 10]. Thus, evidence of a central role of GILZ as an anti-inflammatory agent is lacking and controversial, and requires further exploration, specifically with respect to osteoarthritis (OA), for which intra-articular glucocorticoid injections are used as a symptomatic treatment.

OA is characterized by cartilage breakdown, subchondral bone thickening, osteophyte formation and synovial inflammation. It was first considered to be a degenerative disease caused by aging and mechanical stress. However, obesity has also been identified as a risk factor for digital OA [11] (involving non-weight-bearing joints), suggesting a metabolic influence on OA pathogenesis [12]. The metabolic origin of OA can be partly attributed to the presence of adipokines, such as leptin [13–15], which has a well-established link with OA. For example, the synovial and serum leptin levels have both been correlated with the radiologic score for OA severity [14]; the leptin concentration in synovial fluid has been correlated with body mass index (BMI) [16]; higher leptin expression has been detected in vitro in OA cartilage tissues compared with healthy control tissues [13]; and obesity due to impaired leptin signaling in mice does not lead to OA, in contrast to what is observed in obesity due to overfeeding [15].

In vitro*,* leptin has pro-inflammatory properties; e.g., it induces interleukin-6 (IL-6) expression in synovial fibroblasts [17] and matrix metalloproteinase (MMP) production in human osteoarthritic cartilage [18]. We have previously demonstrated that OA synovial fibroblasts spontaneously produce leptin in vitro [19], suggesting their contribution to the intra-articular leptin level. Expression of leptin and its receptor (Ob-R) are strongly enhanced by glucocorticoids in OA synovial fibroblasts. Furthermore, we have previously demonstrated the involvement of transforming growth factor-β (TGF-β) signaling; prednisolone induces leptin secretion through the ALK1-Smad1/5 pathway, while TGF-β1 suppresses prednisolone-induced leptin secretion through ALK5-Smad2/3 [20].

Glucocorticoids bind to their native receptor, glucocorticoid receptor (GR), and mediate their effects through two different pathways: the “trans-repression” and the “trans-activation” [21]*.* Although this duality is more complex and although these two pathways are less separate than initially described, trans-repression is usually associated with anti-inflammatory effects, and trans-activation is typically correlated with adverse metabolic events. Selective glucocorticoid receptor agonists (SEGRAs) are GR agonists that are known to have a better benefit-risk ratio than glucocorticoids and do not induce adverse metabolic events. They only activate the trans-repression pathway without modulating trans-activation [21]. We previously observed that SEGRA compound A (CpdA), which is known to have a better benefit-risk ratio than glucocorticoids [22], does not induce leptin secretion or Ob-R expression [23]. Therefore, regarding the detrimental role of leptin in OA, we hypothesized that leptin and Ob-R induction could result from glucocorticoid-associated adverse events. However, the underlying mechanisms remain to be elucidated, and the link between GILZ and leptin is unknown.

GR is the native receptor of glucocorticoids. However, glucocorticoids can also act through mineralocorticoid receptor (MR) [24]. Conversely, mineralocorticoids act through GR [25] in addition to their native receptor, MR. Recent studies have suggested that components of metabolic syndrome are associated with abnormal aldosterone physiology [26]*.* Furthermore, leptin secretion is increased after aldosterone exposure in brown adipose tissue [27]. Of interest, GILZ expression is also induced by mineralocorticoids, e.g.*,* its involvement in epithelial channel induction in the kidneys occurs through MR [28].

Therefore, in the present study, we investigated the following issues: (1) whether GILZ expression is also induced by mineralocorticoids in human OA synovial fibroblasts and whether this effect is dependent on either GR or MR; (2) whether GILZ and leptin expression are closely correlated; and (3) whether GILZ plays a central role as an anti-inflammatory agent in this type of cell.

## Methods

### Human synovial fibroblasts and cell culturing

Synovial tissue was obtained from patients with OA during knee replacement surgery. All patients (n = 16) presented with symptomatic knee OA without inflammatory disease or cancer. None of the patients had received oral or intra-articular glucocorticoids for at least one year. The mean patient age was 71 (58–82) years, and the mean BMI was 26.3 (22.2–32.7) kg/m^2^. Seven patients were female (44 %).

Synovial fibroblasts were isolated as previously described [19]. Cells were cultured in DMEM (Cambrex Bio Science, USA) with 10 % FBS (Lonza, Switzerland), L-glutamine (2 mM), streptomycin (100 mg/mL) and penicillin (100 U/mL) (BioWhittaker, USA). A total of 5 × 10^5^ synovial fibroblasts/well were plated in 24-well plates (BD Biosciences, USA). Cells were used at passages 3–7 and were stimulated with the glucocorticoid prednisolone (1 μM, unless stated otherwise), the mineralocorticoid aldosterone (1 and 10 μM, unless stated otherwise), the GR antagonist mifepristone (5 μM), the MR antagonist eplerenone (5 μM), the MR antagonist spironolactone (5 μM) (Sigma-Aldrich, USA), the SEGRA CpdA (1 and 10 μM) (Santa Cruz Biotechnology, USA), TGF-β1 (10 ng/mL) (Gibco-BRL, USA), tumor necrosis factor-α (TNF-α) (10 ng/mL) (Biosource, USA), and MG132 (10 μM) (Alexis Corporation, Switzerland). The proteasome inhibitor MG132 was added to the medium in the last 12 h to enhance GILZ visualization when GILZ was analyzed with western blot.

### Transfection with lentiviruses expressing GILZ and GR small hairpin RNA (shRNA)

Lentiviral vectors were generated by co-transfecting Lenti-X 293 T cells (Clonetech, Belgium) with a pSPAX2 plasmid (Addgene, Plasmid #12260), a VSV-G-encoding vector, a GILZ (TSC22D3) shRNA plasmid (#TRCN0000013793 (GILZ shRNA1), #TRCN0000364625 (GILZ shRNA2) or #TRCN0000369187 (GILZ shRNA3), Sigma-Aldrich, USA), a GR (NR3C1) shRNA plasmid (#TRCN0000245007 (GR shRNA1), #TRCN0000245003 (GR shRNA2) or #TRCN0000245004 (GR shRNA3), Sigma-Aldrich, USA), or a non-target sequence-encoding plasmid (Sigma, Belgium, SHC002). At 72 h post-transfection, viral supernatants were collected, filtered, and concentrated 100 × by ultracentrifugation. Lentiviral vectors were then titrated using a quantitative polymerase chain reaction (PCR) Lentivirus Titration (Titer) Kit (ABM, USA, LV900). A total of 5 × 10^5^ synovial fibroblasts/well were plated in 24-well plates (BD Biosciences, USA) and infected with lentiviruses at a multiplicity of infection (MOI) of 30, unless otherwise indicated. After 72 h of incubation, the medium was removed, and the cells were stimulated.

### Enzyme-linked immunosorbent assay (ELISA)

A commercially available sandwich ELISA kit (R&D Systems, USA) was used to quantify leptin, IL-6, IL-8 and MMP-1 expression in the culture supernatants. All experiments were performed in triplicate.

### Western blotting

Total protein extracts were prepared as described previously [19]. Ob-R (B-3) (sc-8391), GR (41) (sc-136209), GILZ (sc-33780) (Santa Cruz Biotechnology, USA), and glyceraldehyde 3-phosphate dehydrogenase (GAPDH) (Sigma-Aldrich, USA) primary antibodies were used. Western blots were visualized with an anti-mouse or anti-rabbit secondary antibody (Cell Signaling, USA) diluted 1/1000 and enhanced chemiluminescence reagents (GE Healthcare, UK). Western blot band quantification was performed with Image Studio Lite software (USA), and protein expression levels were normalized to the GAPDH level.

### Quantitative reverse transcription PCR (RT-qPCR)

Total RNA was isolated from synovial fibroblasts (either untreated or treated with the indicated concentration of prednisolone or aldosterone for 5 days) and purified using a Nucleospin RNA Kit, with rDNAse included (#740955,). Next, complementary DNA (Cdna) was synthesized by the reverse transcription of 1 μg RNA (in each reaction) with a RevertAid H Minus First Strand cDNA Synthesis Kit (#K1632, Thermo Scientific, Belgium) according to the manufacturer’s instructions. The resulting cDNA was subsequently PCR amplified with a KAPA SYBR FAST detection system (#KK4611, Belgium). Real-time PCR was performed using a LightCycler 480 instrument (Roche, Belgium), and data were analyzed with LC480 software, release 1.5.0 SP4. cDNA dilution curves were generated for each gene and were used to calculate individual real-time PCR efficiencies (*E* = 10^[−1/slope]^). The 2^−ΔΔCT^ method was then used to calculate the relative gene expression levels between the untreated (calibrator sample) and treated synovial fibroblasts. Input amounts were normalized to the *β2-microglobulin* endogenous control gene. All primers were purchased from Eurogentec (Belgium). The following primer sequences were used: *leptin*, 5′- AACCCTGTGCGGATTCTTGT-3′ (forward) and 5′- TCTTGGACTTTTTGGATGGGC-3′ (reverse); *GILZ*, 5′-GCACAATTTCTCCATCTCCTTCTT-3′ (forward) and 5′- TCAGATGATTCTTCACCAGATCCA-3′ (reverse); *β2-microglobulin*, 5′-TTTCATCCATCCGACATTGA-3′ (forward) and 5′-CCAGTCCTTGCTGAAAGACA-3′ (reverse).

### Statistical analysis

Log transformation was applied to all variables to normalize their distribution. Statistical analysis was performed using GraphPad Prism software (version 6) by one-way analysis of variance (ANOVA) for multiple comparisons, followed by Tukey’s post hoc test. The results were considered significant at a *p* value <0.05. Graphs were constructed using the mean ± standard deviation (SD) calculated from three or more independent experimental replicates for the ELISA, RT-qPCR and western blot experiments. Independent experiments were performed using synovial fibroblasts from different patients (n = 3 or more).

## Results

### Prednisolone and aldosterone induced GILZ protein expression through GR in human OA synovial fibroblasts

**Fig. 1:**
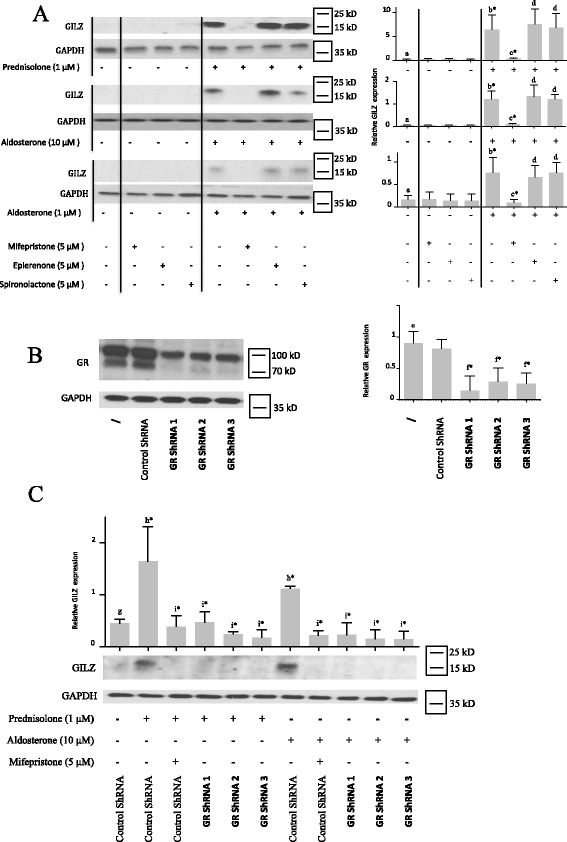
Glucocorticoid-induced leucine zipper (*GILZ*) expression is induced by prednisolone and aldosterone through glucocorticoid receptor (*GR*). **a** Human osteoarthritis (OA) synovial fibroblasts were pre-incubated or not for 1 h with a GR inhibitor (mifepristone) or mineralocorticoid receptor (*MR*) inhibitors (eplerenone and spironolactone) and were then stimulated for 5 days with a glucocorticoid (prednisolone) or a mineralocorticoid (aldosterone). GILZ and glyceraldehyde 3-phosphate dehydrogenase (*GAPDH*) expression in whole-cell extracts were analyzed by western blotting. *Right panels*, quantification results of western blots shown in *left panels*. Protein levels were normalized to GAPDH. Graphs represent mean +/- SD (n = 4 patients). Significance was set at *p* < 0.05. b*Significantly different from *a*; c*significantly different from *b*; *d* not significantly different from *b*. **b**, **c** Synovial fibroblasts were infected with three different lentiviruses expressing GR shRNA or with a non-target control lentivirus and were stimulated or not for 5 days with prednisolone or aldosterone. A GR inhibitor (mifepristone) was used as a positive control for GR inactivation. GILZ, GR, and GAPDH expression in whole-cell extracts were analyzed by western blotting. *Right* (**b**) and *upper* (**c**) *panels*, quantification results of western blots shown in *left panels* and *lower panels*. Protein levels were normalized to GAPDH. Graphs represent mean +/- SD (n = 3 patients). Significance was set at *p* < 0.05. f*Significantly different from *e*; h*significantly different from *g*; i*significantly different from *h. kD* kiloDalton, *shRNA* short hairpin RNA

Human OA synovial fibroblasts were stimulated for 5 days with prednisolone (1 μM) (a glucocorticoid) or aldosterone (1 or 10 μM) (a mineralocorticoid). Western blotting (Fig. 1a) revealed that prednisolone and aldosterone induced GILZ expression in these cells.

Glucocorticoids and mineralocorticoids have affinity for both GR and MR. Therefore, both receptors were investigated to determine which one is involved in GILZ expression. Cells were pre-incubated with a GR inhibitor (5 μM mifepristone) or MR inhibitor (5 μM eplerenone or 5 μM spironolactone) and were then stimulated for 5 days with prednisolone (1 μM) or aldosterone (1 or 10 μM). The GR inhibitor mifepristone strongly reduced both prednisolone- and aldosterone-induced GILZ protein expression (Fig. 1a). In contrast, the MR inhibitors eplerenone and spironolactone did not significantly modulate prednisolone-induced or aldosterone-induced GILZ expression in any experiments (Fig. 1a). These results suggest that mineralocorticoids and glucocorticoids induce GILZ expression through GR but not MR in OA synovial fibroblasts.

To further confirm the involvement of GR in prednisolone-induced and/or aldosterone-induced GILZ expression, we performed shRNA experiments to silence GR. Cells were infected with three different lentiviruses expressing GR shRNA or with a non-target control lentivirus. GR shRNA1, 2 and 3, but not the control shRNA, reduced endogenous GR expression (Fig. 1b). Two bands were observed: the antibody was raised against amino acids 176-289 of GR of human origin. It can therefore recognize both isoforms and probably other GRα transcript splice variants, which could explain the presence of two large bands. Prednisolone-induced and aldosterone-induced GILZ expression was reduced when GR was silenced (Fig. 1c). The GR antagonist mifepristone was used as a positive control to demonstrate GR inactivation. Thus, we confirmed that glucocorticoid-induced and mineralocorticoid-induced GILZ expression was GR-dependent by the silencing of GR.

### Leptin and GILZ were similarly modulated; leptin secretion and Ob-R expression were induced by prednisolone and aldosterone through GR

**Fig. 2:**
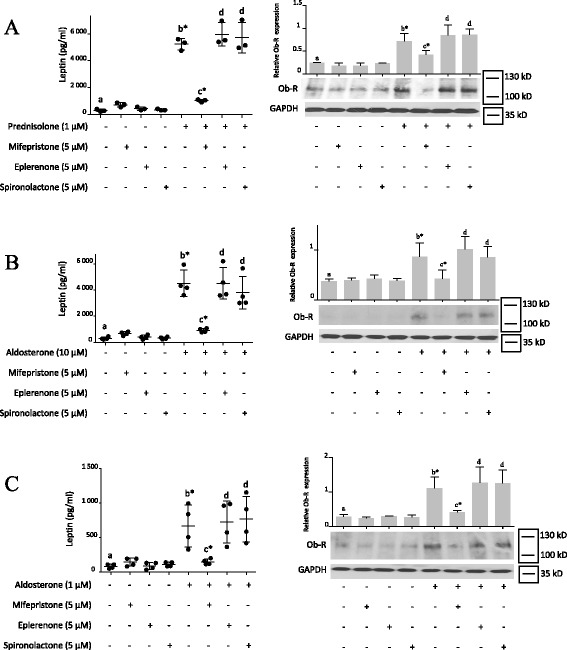
Leptin secretion and leptin receptor (*Ob-R*) expression were induced by prednisolone and aldosterone through glucocorticoid receptor (GR) signaling. Human osteoarthritis (OA) synovial fibroblasts were pre-incubated or not for 1 h with a GR inhibitor (mifepristone) or mineralocorticoid receptor **(**MR) inhibitors (eplerenone and spironolactone) and were then stimulated for 5 days with a glucocorticoid (prednisolone) (**A**) or mineralocorticoid (aldosterone) (**B**, **C**). Leptin expression was measured in cell culture supernatants by ELISA. Ob-R and glyceraldehyde 3-phosphate dehydrogenase (*GAPDH*) expression in whole-cell extracts were analyzed by western blotting. *Upper panels* (*right*), quantification results of western blots shown in *bottom panels*. Proteins levels were normalized to GAPDH. Graphs represent mean +/- SD (n = 3 or 4 patients). Significance set at *p* < 0.05. b*Significantly different from *a*; c*significantly different from *b*; *d* not significantly different from *b. kD* kiloDalton

**Fig. 3:**
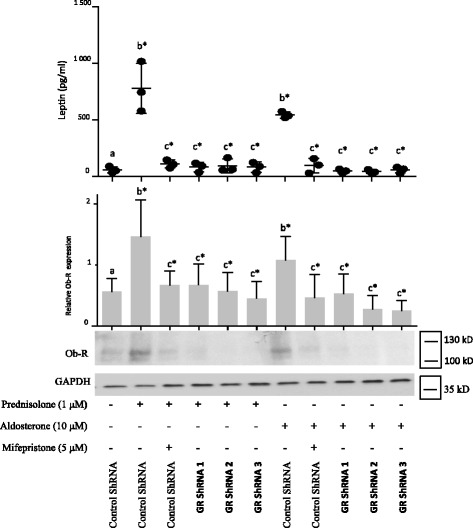
Leptin secretion and leptin receptor (*Ob-R*) expression were induced by prednisolone and aldosterone through glucocorticoid receptor (GR) signaling. Synovial fibroblasts were infected with three different lentiviruses expressing GR short hairpin RNA (*shRNA*) or with a non-target control lentivirus and were stimulated for 5 days with prednisolone or aldosterone. The GR inhibitor mifepristone was used as a positive control for GR inhibition. Leptin expression was measured in the cell culture supernatants by ELISA. Ob-R and glyceraldehyde 3-phosphate dehydrogenase (*GAPDH*) expression were analyzed in whole-cell extracts by western blotting. *Middle panel*, quantification results of western blots shown in the *lower panel*. Protein levels were normalized to GAPDH. Graphs represent mean +/- SD (n = 3 patients). Significance was set at *p* < 0.05. b*Significantly different from *a*; c*significantly different from b*. *kD* kiloDalton

We have previously shown that glucocorticoids enhance leptin secretion and Ob-R expression in synovial fibroblasts ([19] and Fig. 2a: b* vs. a). Similar to prednisolone (Fig. 2a), the mineralocorticoid aldosterone also induced leptin secretion and Ob-R protein expression at 1 μM (Fig. 2b: b* vs. a) and 10 μM (Fig. 2c: b* vs. a). Moreover, using specific inhibitors (a GR inhibitor (mifepristone) and MR inhibitors (eplerenone and spironolactone)), we observed that the prednisolone-induced (Fig. 2a: c* *vs.* b*) and aldosterone-induced (Fig. 2b and c: c* *vs.* b*) leptin secretion and Ob-R expression were GR-dependent but not MR-dependent in all experiments, as observed with GILZ. These results suggest that aldosterone induces leptin secretion and Ob-R expression in OA synovial fibroblasts through GR but not MR, similar to glucocorticoids. The use of GR and MR antagonists did not modulate the endogenous level of leptin secretion or Ob-R expression. Human OA synovial fibroblasts were then infected with three different lentiviruses expressing GR shRNA or with a non-target control lentivirus. Prednisolone-induced and aldosterone-induced leptin secretion and Ob-R expression were abolished when GR was silenced (Fig. 3)*,* confirming the involvement of GR signaling in the induction of these processes.

**Fig. 4:**
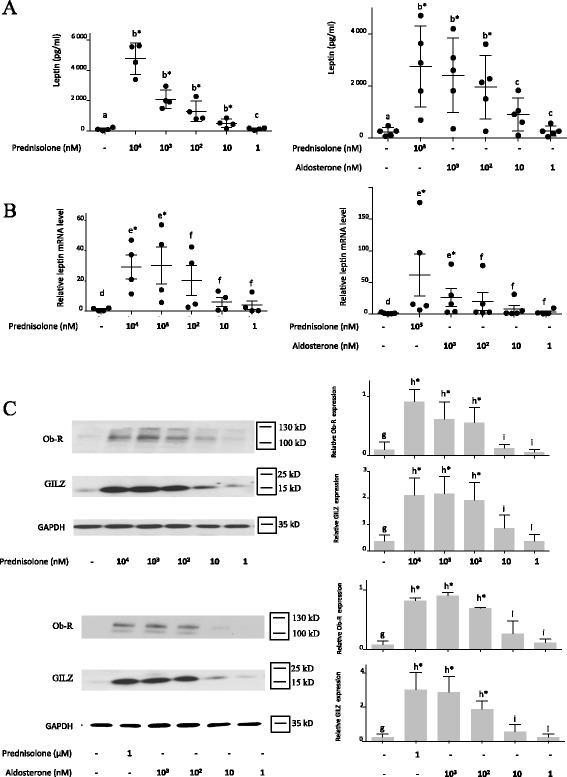
Leptin secretion and leptin receptor (*Ob-R*) and glucocorticoid-induced leucine zipper (*GILZ*) expression were dose-dependent. **a**, **b** Human osteoarthritis (OA) synovial fibroblasts were stimulated for 5 days with increasing concentrations of a glucocorticoid (prednisolone) or mineralocorticoid (aldosterone). Leptin secretion was measured in the cell culture supernatants by ELISA, and leptin messenger RNA (mRNA) expression was measured by RT-qPCR. Graphs represent mean +/- SD (n = 4 or 5 patients). Significance was set at *p* < 0.05. b*Significantly different from *a*; *c* not significantly different from *a*; e*significantly different from *d*; *f* not significantly different from *d*. **c** Human OA synovial fibroblasts were stimulated for 5 days with increasing concentrations of a glucocorticoid (prednisolone) or mineralocorticoid (aldosterone). Ob-R, GILZ and glyceraldehyde 3-phosphate dehydrogenase (*GAPDH*) expression in whole-cell extracts were analyzed by western blotting. *Right panels*, quantification results of western blots shown in the *left panels*. Protein levels were normalized to GAPDH. Graphs represent mean +/- SD (n = 3 patients). Significance was set at *p* < 0.05. h*Significantly different from *g*; *i* not significantly different from *g*

**Fig. 5:**
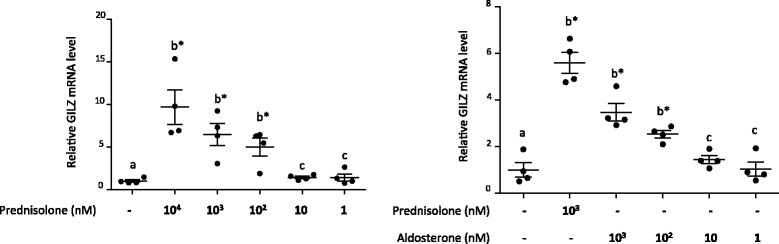
Glucocorticoid-induced leucine zipper (*GILZ*) expression under prednisolone and aldosterone stimulation was dose dependent. Human osteoarthritis (OA) synovial fibroblasts were stimulated for 5 days with increasing concentrations of a glucocorticoid (prednisolone) or mineralocorticoid (aldosterone). GILZ messenger RNA (mRNA) expression was measured by RT-qPCR. Graphs represent mean +/- SD (n = 4 patients). Significance was set at *p* < 0.05. b*Significantly different from *a*; *c* not significantly different from *a*

The induction of leptin by prednisolone and aldosterone was dose-dependent (Fig. 4a and b). A dose response was observed not only for leptin secretion in the cell culture supernatant (as measured by ELISA) (Fig. 4a) but also for leptin messenger RNA (mRNA) expression (as measured by RT-qPCR) (Fig. 4b). The induction of leptin by prednisolone was significant at concentrations of equal to or greater than 10 nM or 1000 nM for ELISA or RT-qPCR, respectively. However, it was not significant at 100 nM or 10 nM for RT-qPCR; although the mean value was higher than that for the control, the variance was high. Leptin induction by aldosterone was significant at concentrations of 100 nM and 1000 nM for ELISA and RT-qPCR, respectively. The induction of Ob-R (Fig. 4c) and GILZ (Fig. 4c for western blot and Fig. 5 for qRT-PCR) by prednisolone and aldosterone was also dose-dependent, with significant induction at a concentration equal to or greater than 100 nM.

**Fig. 6:**
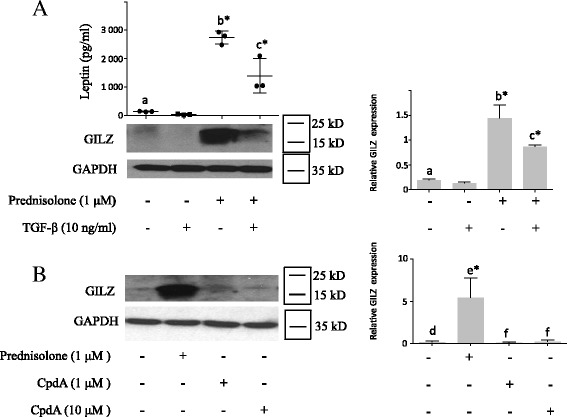
Transforming growth factor-β (*TGF-β*) decreased both prednisolone-induced leptin secretion and glucocorticoid-induced leucine zipper (*GILZ*) expression; Compound A (*CpdA*) did not induce GILZ expression. Human osteoarthritis (OA) synovial fibroblasts were stimulated for 5 days with prednisolone, TGF-β (**A**), or CpdA (**B**). Leptin expression was measured in the cell culture supernatants by ELISA. GILZ and glyceraldehyde 3-phosphate dehydrogenase (*GAPDH*) expression in whole-cell extracts was analyzed by western blotting. *Right panels*, quantification results of western blots shown in the *left panels*. Protein levels were normalized to GAPDH. Graphs represent mean +/- SD (n = 3 patients). b*Significantly different from *a*; c*significantly different from *b*; e*significantly different from *d*; *f* not significantly different from *d*

TGF-β did not induce GILZ expression, and it decreased prednisolone-induced GILZ expression (Fig. 6a), which is in accordance with our previous results demonstrating that TGF-β does not induce leptin secretion and that it decreases glucocorticoid-induced leptin secretion [20]. Moreover, the selective GR agonist CpdA, which does not induce leptin expression [23], also does not induce GILZ expression (Fig. 6b). Taken together, these results indicate that leptin and GILZ have similar expression profiles, suggesting correlation between their expression.

### GILZ was involved in prednisolone-induced and aldosterone-induced leptin and Ob-R expression

**Fig. 7:**
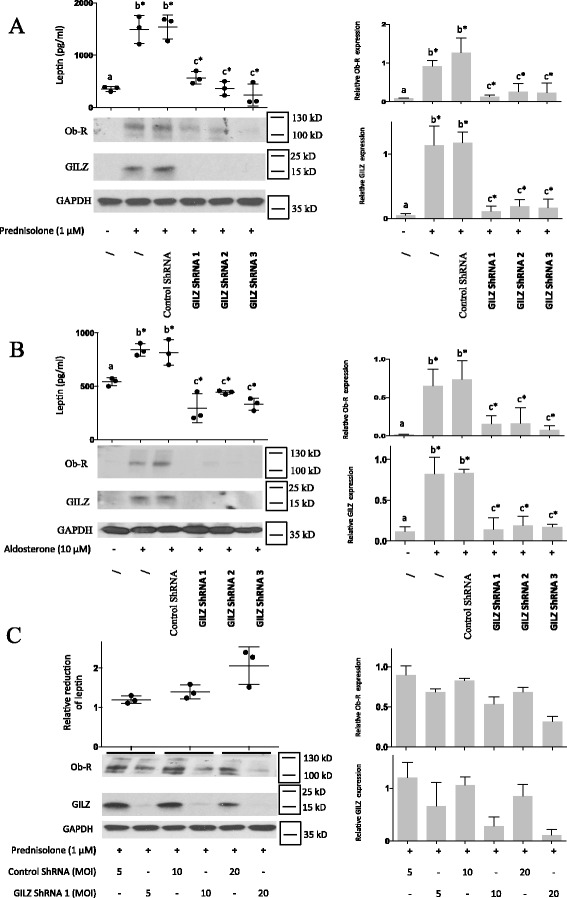
Glucocorticoid-induced leucine zipper (*GILZ*) silencing inhibited glucocorticoid-induced and mineralocorticoid-induced leptin and leptin receptor (*Ob-R*) expression. Human osteoarthritis (OA) synovial fibroblasts were infected with three different lentiviruses expressing GILZ short hairpin RNA (*shRNA*) or with a control lentivirus (multiplicity of infection (MOI) 30 for **a** and **b**; MOI 5, 10 and 20 for **c**). After 72 h, the cells were stimulated for 5 days with a glucocorticoid (1 μM prednisolone) or mineralocorticoid (10 μM aldosterone). Leptin expression was measured in the cell culture supernatants by ELISA. Ob-R, GILZ and glyceraldehyde 3-phosphate dehydrogenase (*GAPDH*) expression in whole-cell extracts was analyzed by western blotting. *Right panels*, quantification results of western blots shown in the *left panels*. Protein levels were normalized to GAPDH. Graphs represent mean +/- SD (n = 5 patients). b*Significantly different from *a*; c*significantly different from *b. kD* kiloDalton

**Fig. 8:**
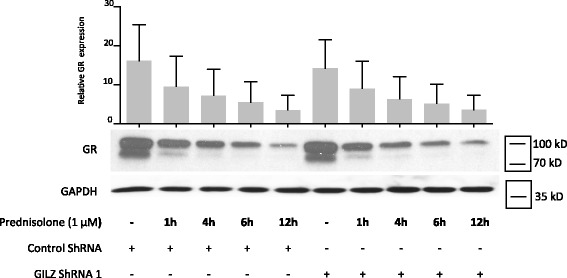
Glucocorticoid-induced leucine zipper (GILZ) silencing did not alter prednisolone-induced glucocorticoid receptor (*GR*) degradation. Human osteoarthritis (OA) synovial fibroblasts were infected with a lentivirus expressing GILZ short hairpin RNA (*shRNA*) or with a control lentivirus. After 72 h, the cells were stimulated for 1, 4, 6, or 12 h with a glucocorticoid (1 μM prednisolone). Leptin expression was measured in the cell culture supernatants by ELISA. GILZ and glyceraldehyde 3-phosphate dehydrogenase (*GAPDH*) expression in whole-cell extracts was analyzed by western blotting. *Upper panel*, quantification results of western blot shown in the *lower panel*. Protein levels were normalized to GAPDH. Graph represents mean +/- SD (n = 3) patients. *kD* kiloDalton

ShRNA experiments for the silencing of GILZ expression were performed to determine whether GILZ is involved in prednisolone- and/or aldosterone-induced leptin secretion. Human OA synovial fibroblasts were infected with three different lentiviruses expressing GILZ shRNA or with a non-target control lentivirus. After 72 h of incubation, the medium was removed, and the cells were stimulated with prednisolone (1 μM) or aldosterone (1 or 10 μM). GILZ shRNA reduced GILZ expression following prednisolone (Fig. 7a) or aldosterone (Fig. 7b) stimulation. Downregulation of GILZ expression resulted in significant decreases in prednisolone-induced (Fig. 7a) and aldosterone-induced (Fig. 7b) leptin and Ob-R expression compared with the controls. These decreases were correlated with the shRNA MOI and with the degree of GILZ silencing (Fig. 7c). GILZ silencing did not alter GR expression or prednisolone-induced GR degradation (Fig. 8).

### GILZ inhibition did not alter the anti-inflammatory properties of prednisolone

**Fig. 9:**
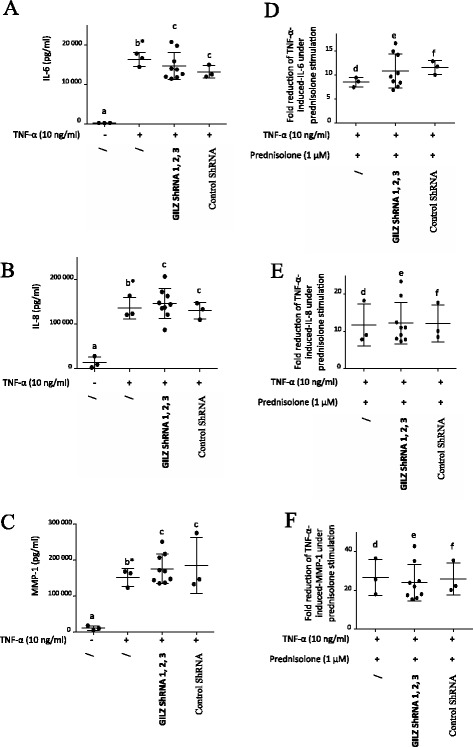
Glucocorticoid-induced leucine zipper (*GILZ*) inhibition did not alter the capacity for prednisolone to reduce IL-6, IL-8 and matrix metalloproteinase-1 (*MMP-1*) production. Human osteoarthritis (OA) synovial fibroblasts were infected with three different lentiviruses expressing GILZ short hairpin RNA (*shRNA*) or with a control lentivirus (one sample was infected with one shRNA and the three results were pooled). Cells were pre-incubated (**D**, **E**, **F**) or not (**A**, **B**, **C**) for 1 h with prednisolone (1 μM) and were then stimulated or not with TNF-α (10 ng/mL) for 12 h. The IL-6, IL-8 and MMP-1 levels in the cell culture supernatants were measured by ELISA. Graphs represent mean +/- SD (n = 3 patients). Fold reductions in IL-6, IL-8, and MMP-1 induced by TNF-α were measured by comparing the levels in the presence or not of prednisolone (1 μM). b*Significantly different from *a*; *c* not significantly different from *b*; *e f* not significantly different from *d*

Synovial fibroblasts spontaneously produced the pro-inflammatory cytokines IL-6, IL-8 and MMP-1, and cell stimulation by TNF-α enhanced the secretion of these cytokines (Fig. 9a-c). Human OA synovial fibroblasts were infected with three different lentiviruses expressing GILZ shRNA or with a non-target control lentivirus. Cells were pre-incubated (Fig. 9d-f) or not (Fig. 9a-c) for 1 h with prednisolone (1 μM) and were then stimulated or not with TNF-α (10 ng/mL) for 12 h. GILZ-shRNA did not significantly alter TNF-α-induced IL-6, IL-8 and MMP-1 production (Fig. 9a-c). Moreover, GILZ inhibition did not alter the capacity for prednisolone to reduce the TNF-α-induced production of these cytokines (Fig. 9d-f). These results suggest that GILZ is not an essential mediator of the anti-inflammatory activities of glucocorticoids in OA synovial fibroblasts.

## Discussion

Glucocorticoids are widely used by rheumatologists, either to treat a flare in inflammatory rheumatic diseases or to reduce pain and swelling through intra-articular injection into joints in OA [29]. Unfortunately, they also contribute to adverse events, such as diabetes mellitus and osteoporosis. Moreover, glucocorticoids enhance a catabolic reaction leading to the degradation of cartilage [30], and they suppress matrix protein markers of chondrogenic differentiation [31].

GILZ is an intracellular protein induced by glucocorticoids and is mainly present in immune cells [32–34]*.* It can also be induced by mineralocorticoids, as observed in kidney cells [28]. Beaulieu et al. have observed GILZ expression in the synovium of patients with rheumatoid arthritis and in cultured rheumatoid arthritis synovial fibroblasts and have found that its expression is enhanced by dexamethasone and significantly reduced by mifepristone, suggestive of GR involvement [8]. Our work extends the presence of GILZ to human OA synovial fibroblasts and demonstrates for the first time that both the glucocorticoid prednisolone and the mineralocorticoid aldosterone induce GILZ expression in these cells in a dose-dependent and GR-dependent process, at the protein and the mRNA level.

We propose a novel role of GILZ in contributing to corticoid-induced leptin and Ob-R expression in OA synovial fibroblasts. Indeed, we have previously reported that human OA synovial fibroblasts produce leptin (a pro-inflammatory adipokine involved in OA pathogenesis) and its receptor, Ob-R, both spontaneously and after stimulation with glucocorticoids [19]. We have also previously demonstrated that leptin induction occurs independently of any adipogenesis-related processes by performing oil red staining and that it occurs in the absence of adipogenic mediators [19]. Among adipokines, leptin is of particular interest in metabolic OA, as we did not detect any endogenous or glucocorticoid-induced secretion of resistin or adiponectin in OA synovial fibroblasts [23]. The deleterious contribution of leptin to the pathogenesis of metabolic OA [15] indicates that leptin and Ob-R expression in synovial fibroblasts contribute to the adverse metabolic events caused by glucocorticoids. GILZ, leptin and Ob-R protein expression are closely correlated, as demonstrated by the significant reductions in leptin and Ob-R expression following GILZ silencing.

Moreover, we previously reported that TGF-β reduced the prednisolone-induced leptin secretion through ALK5-Smad2/3 [20]. In this work, we observed that TGF-β did not induce GILZ and reduced the prednisolone-induced GILZ expression, which is coherent with our previous results. This is also coherent with previous works in human bronchial epithelial cells, where TGF-β1 impaired the glucocorticoid trans-activation, did not induce GILZ mRNA and even reduced dexamethasone-induced GILZ expression also through ALK5-Smad2/3 [35].

GILZ is primarily described as a mediator of the anti-inflammatory activities of glucocorticoids in immune-related cells [32–34]. GILZ overexpression has significant anti-inflammatory effects in the treatment of collagen-induced arthritis [9, 10]. However, in the current study, GILZ depletion in OA synovial fibroblasts did not alter either the TNF-α-induced pro-inflammatory protein levels (IL-6, IL-8, and MMP-1) or the capacity for glucocorticoids to downregulate the TNF-α-induced production of IL-6, IL-8, and MMP-1, supporting the notion that GILZ is not significantly involved in the anti-inflammatory regulation of glucocorticoids in OA. GILZ downregulation did not modulate GR expression or GR downregulation. A lack of inflammatory modulation in synovial cells following GILZ downregulation has also been reported in mouse models of collagen-induced arthritis [9] and in human umbilical venous endothelial cells [10]*.* Last, although Beaulieu et al. [8] have previously shown that GILZ overexpression significantly inhibits the production of key pro-inflammatory cytokines, such as IL-6 and IL-8, in rheumatoid arthritis synovial fibroblasts, it has no effect on a panel of additional pro-inflammatory cytokines, such as IL-1β, TNF-α, and IL-12p70, which are produced by the same cells. Thus, involvement of GILZ in the inflammatory process appears to be cell-dependent.

In this study, we showed that the SEGRA CpdA (which activates only the trans-repression pathway and has a better benefit-risk ratio than glucocorticoids [22]) did not induce GILZ expression. This finding is consistent with our previous study showing that CpdA does not induce leptin secretion or Ob-R expression and that it exhibits similar anti-inflammatory effects to classical glucocorticoids [23], positioning the leptin secretion in the trans-activation pathway. While GILZ is not necessary for the glucocorticoid anti-inflammatory effect and is involved in leptin secretion, absence of GILZ induction by CpdA seems coherent. This result is also in agreement with Drebert et al., who have demonstrated a lack of GILZ induction under CpdA treatment in colon-cancer-derived myofibroblasts [36] and with Gavrila et al. using airway smooth muscle cells [37]. Moreover, other authors have demonstrated that CpdA does not induced DUSP1, which is another actor in the glucocorticoid trans-activation [38].

From a metabolic point of view, it can be hypothesized that GILZ induction is deleterious in OA because it induces the adipokine leptin. Additional examples of the involvement of GILZ in glucocorticoid-associated adverse events have been reported. Dexamethasone and GILZ have been shown to inhibit the repair of respiratory epithelial cells [39]. In addition, the long-term use of glucocorticoids has been demonstrated to have anti-myogenic effects due to the presence of GILZ as an effector [40].

In the present study, aldosterone, a mineralocorticoid, was also found to be a significant stimulator of GILZ, leptin, and Ob-R expressions. These results are in accordance with those of previous studies showing an in vitro increase in the leptin mRNA level in brown adipose tissue after aldosterone exposure [27] and higher circulating leptin levels in patients with primary hyperaldosteronism [41]. Aldosterone is known for its association with a bad metabolic profile (i.e., its association with the development of metabolic syndrome in humans) [42, 43] and for its aggravation of glucose intolerance by high fructose in rats [44]. It is also present in the synovial fluid of patients with OA [45]. Therefore, targeting the aldosterone pathway could be promising for the treatment of OA.

Corticoid concentrations in synovial fluid have not been well-described. With regard to glucocorticoids, human OA synovial fluid has been reported to contain 125 nM cortisol [45]. Significant induction of leptin, Ob-R, and GILZ expression has been observed at this concentration by prednisolone (a synthetic cortisol compound). Accordingly, the significant effect of glucocorticoids on leptin production in the joints in OA may be clinically relevant, even in the absence of exogenous glucocorticoid administration. However, the mineralocorticoid levels are 1000-fold decreased in biologic fluids compared to the glucocorticoid levels. Although 1 μM aldosterone has been used in studies with mechanistic models [25], the physiological concentration is approximately 100 pM in OA synovial fluid [45], which was not sufficient to induce leptin or Ob-R expression in our model. Therefore, we cannot affirm that leptin and GILZ induction by aldosterone is clinically relevant. However, the use of 1 μM and 10 μM aldosterone in the current study confirmed the focus of our mechanistic model on the role of GILZ in leptin expression.

The influence of aldosterone on GILZ, leptin and Ob-R expressions is also GR-dependent; indeed, their induction remains unchanged in the presence of specific MR inhibitors, whereas it is abolished in the presence of specific GR inhibitors or after GR silencing. Lee et al. have observed that GR silencing blocks leptin induction by cortisol in human adipocytes, in contrast with MR silencing [46]*.* Leminen et al. have demonstrated in vivo downregulation of the expression of circulating leptin in humans with the GR inhibitor mifepristone [47]. However, the regulation of leptin by aldosterone appears to be tissue-dependent, and it requires further clarification. In mice with impaired leptin receptor signaling, MR blockade by eplerenone administration in vivo has been shown to reduce leptin mRNA expression in retroperitoneal adipose tissue [48].

## Conclusion

**Fig. 10:**
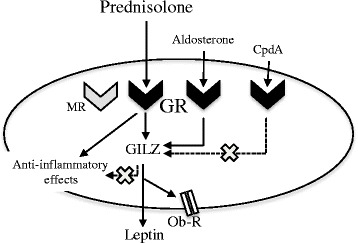
Absence of glucocorticoid-induced leucine zipper (*GILZ*) prevented the corticoid-induced leptin and leptin receptor (*Ob-R*) expression without modulating the anti-inflammatory properties of glucocorticoids. Prednisolone and aldosterone induced GILZ expression in OA synovial fibroblasts through glucocorticoid receptor (*GR*) but not mineralocorticoid receptor (*MR*) activation, whereas Compound A (*CpdA*) did not. Similar effects on leptin secretion and Ob-R expression were observed. Thus, GILZ was involved in prednisolone-induced and aldosterone-induced leptin secretion and Ob-R expression. In addition, GILZ inhibition did not alter the anti-inflammatory action of prednisolone

In conclusion, we describe a new role for GILZ in the corticoid-induced leptin and Ob-R expression in OA synovial fibroblasts. Absence of GILZ prevents corticoid-induced leptin secretion and Ob-R expression without modulating the anti-inflammatory properties of glucocorticoids in OA synovial fibroblasts (Fig. 10). Regarding the deleterious involvement of leptin in OA pathogenesis, the use of GR agonists that do not activate GILZ pathways when using glucocorticoids should be evaluated.

## Abbreviations

BMI: body mass index
CpdA: compound A
DMEM: Dulbecco’s modified Eagle’s medium
ELISA: enzyme-linked immunosorbent assay
FBS: fetal bovine serum
GAPDH: glyceraldehyde 3-phosphate dehydrogenase
GILZ: glucocorticoid-induced leucine zipper
GR: glucocorticoid receptor
IL: interleukin
MMP: matrix metalloproteinase
MOI: multiplicity of infection
MR: mineralocorticoid receptor
mRNA: messenger RNA
OA: osteoarthritis
Ob-R: leptin receptor
RT-qPCR: quantitative reverse transcription PCR
SD: standard deviation
SEGRA: selective glucocorticoid receptor agonist
shRNA: small hairpin RNA
TGF-β: transforming growth factor-β
TNF-α: tumor necrosis factor-α

## References

[1] Yang N, Zhang W, Shi XM (2008). Glucocorticoid-induced leucine zipper (GILZ) mediates glucocorticoid action and inhibits inflammatory cytokine-induced COX-2 expression. J Cell Biochem.

[2] Yang YH, Aeberli D, Dacumos A, Xue JR, Morand EF (2009). Annexin-1 regulates macrophage IL-6 and TNF via glucocorticoid-induced leucine zipper. J Immunol.

[3] Eddleston J, Herschbach J, Wagelie-Steffen AL, Christiansen SC, Zuraw BL (2007). The anti-inflammatory effect of glucocorticoids is mediated by glucocorticoid-induced leucine zipper in epithelial cells. J Allergy Clin Immunol.

[4] Esposito E, Bruscoli S, Mazzon E (2012). Glucocorticoid-induced leucine zipper (GILZ) over-expression in T lymphocytes inhibits inflammation and tissue damage in spinal cord injury. Neurotherapeutics.

[5] Luz-Crawford P, Tejedor G, Mausset-Bonnefont AL (2015). Gilz governs the therapeutic potential of mesenchymal stem cells by inducing a switch from pathogenic to regulatory Th17 cells. Arthritis Rheum.

[6] Frodl T, Carballedo A, Frey EM (2014). Expression of glucocorticoid inducible genes is associated with reductions in cornu ammonis and dentate gyrus volumes in patients with major depressive disorder. Dev Psychopathol.

[7] Bruscoli S, Velardi E, Di Sante M (2012). Long glucocorticoid-induced leucine zipper (L-GILZ) protein interacts with ras protein pathway and contributes to spermatogenesis control. J Biol Chem.

[8] Beaulieu E, Ngo D, Santos L (2010). Glucocorticoid-induced leucine zipper is an endogenous antiinflammatory mediator in arthritis. Arthritis Rheum.

[9] Ngo D, Beaulieu E, Gu R (2013). Divergent effects of endogenous and exogenous glucocorticoid-induced leucine zipper in animal models of inflammation and arthritis. Arthritis Rheum.

[10] Cheng Q, Fan H, Ngo D (2013). GILZ overexpression inhibits endothelial cell adhesive function through regulation of NF-kappaB and MAPK activity. J Immunol.

[11] Oliveria SA, Felson DT, Cirillo PA, Reed JI, Walker AM (1999). Body weight, body mass index, and incident symptomatic osteoarthritis of the hand, hip, and knee. Epidemiology.

[12] Yoshimura N, Muraki S, Oka H (2012). Accumulation of metabolic risk factors such as overweight, hypertension, dyslipidaemia, and impaired glucose tolerance raises the risk of occurrence and progression of knee osteoarthritis: a 3-year follow-up of the ROAD study. Osteoarthritis and cartilage/OARS, Osteoarthritis Research Society.

[13] Dumond H, Presle N, Terlain B (2003). Evidence for a key role of leptin in osteoarthritis. Arthritis Rheum.

[14] Stannus OP, Cao Y, Antony B (2015). Cross-sectional and longitudinal associations between circulating leptin and knee cartilage thickness in older adults. Ann Rheum Dis.

[15] Griffin TM, Huebner JL, Kraus VB, Guilak F (2009). Extreme obesity due to impaired leptin signaling in mice does not cause knee osteoarthritis. Arthritis Rheum.

[16] Vuolteenaho K, Koskinen A, Moilanen T, Moilanen E (2012). Leptin levels are increased and its negative regulators, SOCS-3 and sOb-R are decreased in obese patients with osteoarthritis: a link between obesity and osteoarthritis. Ann Rheum Dis.

[17] Yang WH, Liu SC, Tsai CH (2013). Leptin induces IL-6 expression through OBRl receptor signaling pathway in human synovial fibroblasts. PLoS One.

[18] Koskinen A, Vuolteenaho K, Nieminen R, Moilanen T, Moilanen E (2011). Leptin enhances MMP-1, MMP-3 and MMP-13 production in human osteoarthritic cartilage and correlates with MMP-1 and MMP-3 in synovial fluid from OA patients. Clin Exp Rheumatol.

[19] Relic B, Zeddou M, Desoroux A, Beguin Y, de Seny D, Malaise MG (2009). Genistein induces adipogenesis but inhibits leptin induction in human synovial fibroblasts. Laboratory investigation; a journal of technical methods and pathology.

[20] Zeddou M, Relic B, Malaise O (2012). Differential signalling through ALK-1 and ALK-5 regulates leptin expression in mesenchymal stem cells. Stem Cells Dev.

[21] Sundahl N, Bridelance J, Libert C, De Bosscher K, Beck IM (2015). Selective glucocorticoid receptor modulation: new directions with non-steroidal scaffolds. Pharmacol Ther.

[22] Schacke H, Berger M, Rehwinkel H, Asadullah K (2007). Selective glucocorticoid receptor agonists (SEGRAs): novel ligands with an improved therapeutic index. Mol Cell Endocrinol.

[23] Malaise O, Relic B, Quesada-Calvo F (2014). Selective glucocorticoid receptor modulator compound A, in contrast to prednisolone, does not induce leptin or the leptin receptor in human osteoarthritis synovial fibroblasts. Rheumatology.

[24] Sun B, Chamarthi B, Williams JS (2012). Different polymorphisms of the mineralocorticoid receptor gene are associated with either glucocorticoid or mineralocorticoid levels in hypertension. J Clin Endocrinol Metab.

[25] Ren R, Oakley RH, Cruz-Topete D, Cidlowski JA (2012). Dual role for glucocorticoids in cardiomyocyte hypertrophy and apoptosis. Endocrinology.

[26] Takahashi K, Murase T, Takatsu M (2015). Roles of oxidative stress and the mineralocorticoid receptor in cardiac pathology in a rat model of metabolic syndrome. Nagoya J Med Sci.

[27] Kraus D, Jager J, Meier B, Fasshauer M, Klein J (2005). Aldosterone inhibits uncoupling protein-1, induces insulin resistance, and stimulates proinflammatory adipokines in adipocytes. Horm Metab Res.

[28] Ueda K, Fujiki K, Shirahige K (2014). Genome-wide analysis of murine renal distal convoluted tubular cells for the target genes of mineralocorticoid receptor. Biochem Biophys Res Commun.

[29] Bellamy N, Campbell J, Robinson V, Gee T, Bourne R, Wells G. Intraarticular corticosteroid for treatment of osteoarthritis of the knee. Cochrane Database Syst Rev. 2006;(2):CD005328. 10.1002/14651858.CD005328.pub2 16625636

[30] Celeste C, Ionescu M, Robin Poole A, Laverty S (2005). Repeated intraarticular injections of triamcinolone acetonide alter cartilage matrix metabolism measured by biomarkers in synovial fluid. J Orthop Res.

[31] Fubini SL, Todhunter RJ, Burton-Wurster N, Vernier-Singer M, MacLeod JN (2001). Corticosteroids alter the differentiated phenotype of articular chondrocytes. J Orthop Res.

[32] Riccardi C, Bruscoli S, Ayroldi E, Agostini M, Migliorati G (2001). GILZ, a glucocorticoid hormone induced gene, modulates T lymphocytes activation and death through interaction with NF-kB. Adv Exp Med Biol.

[33] Ayroldi E, Migliorati G, Bruscoli S (2001). Modulation of T-cell activation by the glucocorticoid-induced leucine zipper factor via inhibition of nuclear factor kappaB. Blood.

[34] Berrebi D, Bruscoli S, Cohen N (2003). Synthesis of glucocorticoid-induced leucine zipper (GILZ) by macrophages: an anti-inflammatory and immunosuppressive mechanism shared by glucocorticoids and IL-10. Blood.

[35] Keenan CR, Mok JS, Harris T, Xia Y, Salem S, Stewart AG (2014). Bronchial epithelial cells are rendered insensitive to glucocorticoid transactivation by transforming growth factor-beta1. Respir Res.

[36] Drebert Z, Bracke M, Beck IM (2015). Glucocorticoids and the non-steroidal selective glucocorticoid receptor modulator, compound A, differentially affect colon cancer-derived myofibroblasts. J Steroid Biochem Mol Biol.

[37] Gavrila A, Chachi L, Tliba O, Brightling C, Amrani Y (2015). Effect of the plant derivative Compound A on the production of corticosteroid-resistant chemokines in airway smooth muscle cells. Am J Respir Cell Mol Biol.

[38] Reber LL, Daubeuf F, Plantinga M (2012). A dissociated glucocorticoid receptor modulator reduces airway hyperresponsiveness and inflammation in a mouse model of asthma. J Immunol.

[39] Liu J, Zhang M, Niu C (2013). Dexamethasone inhibits repair of human airway epithelial cells mediated by glucocorticoid-induced leucine zipper (GILZ). PLoS One.

[40] Bruscoli S, Donato V, Velardi E (2010). Glucocorticoid-induced leucine zipper (GILZ) and long GILZ inhibit myogenic differentiation and mediate anti-myogenic effects of glucocorticoids. J Biol Chem.

[41] Iacobellis G, Petramala L, Cotesta D (2010). Adipokines and cardiometabolic profile in primary hyperaldosteronism. J Clin Endocrinol Metab.

[42] Musani SK, Vasan RS, Bidulescu A (2013). Aldosterone, C-reactive protein, and plasma B-type natriuretic peptide are associated with the development of metabolic syndrome and longitudinal changes in metabolic syndrome components: findings from the Jackson Heart Study. Diabetes Care.

[43] Cooper JN, Fried L, Tepper P (2013). Changes in serum aldosterone are associated with changes in obesity-related factors in normotensive overweight and obese young adults. Hypertens Res.

[44] Sherajee SJ, Rafiq K, Nakano D (2013). Aldosterone aggravates glucose intolerance induced by high fructose. Eur J Pharmacol.

[45] Rovensky J, Kvetnansky R, Radikova Z (2005). Hormone concentrations in synovial fluid of patients with rheumatoid arthritis. Clin Exp Rheumatol.

[46] Lee MJ, Fried SK (2014). The glucocorticoid receptor, not the mineralocorticoid receptor, plays the dominant role in adipogenesis and adipokine production in human adipocytes. Int J Obes (Lond).

[47] Leminen R, Raivio T, Ranta S (2005). Late follicular phase administration of mifepristone suppresses circulating leptin and FSH - mechanism(s) of action in emergency contraception?. Eur J Endocrinol.

[48] Guo C, Ricchiuti V, Lian BQ (2008). Mineralocorticoid receptor blockade reverses obesity-related changes in expression of adiponectin, peroxisome proliferator-activated receptor-gamma, and proinflammatory adipokines. Circulation.

